# Breast cancer in Iran: need for greater women awareness of warning signs and effective screening methods

**DOI:** 10.1186/1447-056X-7-6

**Published:** 2008-12-20

**Authors:** Ali Montazeri, Mariam Vahdaninia, Iraj Harirchi, Amir Mahmood Harirchi, Akram Sajadian, Fatemeh Khaleghi, Mandana Ebrahimi, Shahpar Haghighat, Soghra Jarvandi

**Affiliations:** 1 Iranian Institute for Health Sciences Research (IHSR), ACECR, Tehran, Iran; 2 Tehran University of Medical Sciences, Tehran, Iran; 3 University of Social Welfare and Rehabilitation Sciences, Tehran, Iran; 4 Iranian Centre for Breast Cancer (ICBC), ACECR, Tehran, Iran

## Abstract

**Background:**

Breast cancer remains an important public health problem. This study aimed to investigate about female knowledge of breast cancer and self-reported practice of breast self-examination in Iran.

**Methods:**

This was a population-based survey carried out in Tehran, Iran. Data were collected via a structured questionnaire containing 15 questions on demographic status, history of personal and family breast problems, subjective knowledge about breast cancer covering its symptoms, the screening methods and practice of breast self-examination (BSE). A trained female nurse interviewed each respondent. Analysis included descriptive statistics and the Chi-squared test where necessary.

**Results:**

A total of 1402 women were interviewed. The mean age of respondents was 43.4 (SD = 14.4) years; most were married (85%), and without any personal (94%) and family history (90%) of breast problems. It was found that 64% of the respondents were familiar with breast cancer and 61% (n = 851) believed that 'the disease is relatively common among women in Iran'. Most women (44%) perceived a painless mass as a breast cancer symptom. Overall, 61% of the respondents stated that they knew about breast cancer screening programs and most indicated that electronic media (television 34% and radio 14%) were their source of information. Only 17% of women said that 'they were conducting regular breast self-examination'. The main reason for women not doing breast self-examination was due to the fact that they did not know how to do it (64%). The findings indicated that performing breast self-examination is significantly related to: age, marital status, education, knowledge of breast cancer and knowledge about breast cancer screening programs (p < 0.05), but not to personal (P = 0.2) and family (P = 0.7) history of breast problems.

**Conclusion:**

This descriptive study provides useful information that could be utilized by both researchers and those involved in public health programmes. The findings indicated that the women awareness of breast cancer warning signs (painless lump, retraction of nipple, and bloody discharge) and effective screening methods i.e. clinical examination, and mammography were very inadequate. Thus, health education programmes to rectify the lack of women awareness is urgently needed. Indeed the focus of primary health care providers should be to raise awareness about breast care among women and to encourage them to report any unusual changes in their breasts to their family or care physicians.

## Background

Breast cancer is one of the most common cancers among females worldwide. Global statistics show the annual incidence of breast cancer is increasing and this is occurring more rapidly in countries with a low incidence rate of breast cancer [1,2]. It has been reported that each year over 1.15 million women worldwide are diagnosed with breast cancer and 502,000 die from the disease [3]. In Iran the incidence of the disease is rising, patients present with advanced stage of disease and they are relatively younger (about 10 years) than their western counterparts [4,5]. The latest formal information on age-specific incidence rate of female breast cancer per 100,000 population in Iran is presented in Table 1[6].

**Table 1:** Age-specific incidence rate of female breast cancer per 100.000 population in Iran in 2005–2006 (n = 5981)

	**Incidence rate**
**Age groups (years)**	
15–19	0.02
20–24	0.78
25–29	5.91
30–34	14.74
35–39	33.14
40–44	47.90
45–49	74.03
50–54	74.31
55–59	69.28
60–64	69.09
65–69	48.58
70–74	43.71
75–79	44.53
80–84	58.45
≥ 85	22.09
Crude rate	17.44
Age specific rate (weighted)	23.16

**Type of breast cancer**	**%**
Infiltrating ductal carcinoma	80
Infiltrating lobular carcinoma	5.9
Medullary carcinoma	2.6

Early detection of breast cancer plays the leading role in reducing mortality rates and improving the patients' prognosis [7]. The recommended screening methods for early detection of this fatal disease are: mammography, clinical breast examination and breast self-examination (BSE).

Mammography is an expensive modality for screening and needs several requisites including logistic and professional manpower before its implementation. Randomized trials comparing mammography with no mammography screening found that at best women might benefit a 15% relative risk reduction in mortality from mammography [8]. In addition it has been shown that for women under the age of 50 years mammography screening is ineffective [9].

Clinical breast examination is relatively simple and inexpensive but the exact benefit of this screening modality in reducing mortality is yet to be established. It is argued that in diagnosis of breast cancer by screening the shift should be to the point that will cost least both in human and financial terms and be effective in reducing mortality, and that clinical breast examination would be able to fulfill this [10]. However, it has been shown that clinical breast examination could only detect about 60% of breast cancers detected by mammography as well as some breast cancers not detected by mammography [11]. Recent estimates suggest that clinical breast examination has a sensitivity of about 54% and specificity of about 94% [12].

Unlike to mammography and clinical breast examination, BSE is simple, inexpensive, low in technology, teaching is possible to both health professionals and women and more importantly raises awareness about breast cancer in women. It is argued that in many countries, especially in developing countries, BSE may be the only realistic approach to the early detection of breast cancer [10]. However there is no clear evidence to support the efficacy of performing routine BSE in early detection and cause specific mortality due to breast cancer. While a number of studies have found that BSE has improved early detection and reduced mortality [13,14], data from a randomized trial in Russia has suggested that there is no significant difference in those who perform BSE and those who do not in terms of the size of primary tumor and the incidence of metastatic lesion lymph nodes at detection [15,16]. Also the Shanghai randomized trial demonstrated no significant stage shift or mortality reduction from breast cancer after 5 to 10 years of follow-up in the BSE group compared to controls [17,18]. Similarly a literature review on the topic indicated no benefit from routine BSE instruction as a screening tool for breast cancer [19]. However, despite continuous debate about the efficacy of BSE [20,21], it seems that breast self-examination not as a public health policy but as a preventive measure remains a method of choice for early detection of breast cancer in developing countries. Resource constraints in low and middle-income regions lead to limited application of established guidelines for breast health care in developed countries [22].

To summarize it should be noted that in fact none of the above modalities for breast cancer screening could be regarded as the best method for early detection and mortality reduction. These approaches have their own potential benefits and harms. Thus, at present the emphasis is to raise breast cancer awareness among women to overcome ever-increasing burden of the disease. It appears that overall the best way to save women's life is to make them aware of the potential benefits and harms of these approaches and to raise their knowledge about the warning signs of breast cancer. At present in addition to public health professionals, even oncologists advise breast awareness over routine breast self-examination [23]. However, one should not confuse between breast cancer awareness and breast self-examination since these are not the same. Breast cancer awareness can be defined as 'a woman becoming familiar with her own breasts and the way that they will change throughout her life' [24].

Breast cancer awareness programmes have been very successful at several grounds including creation of greater compliance with breast cancer prevention and screening strategies [25]. However, women with different cultures vary in their views about breast cancer and about preventive strategies [26]. This paper reports data from Iran (a culturally diverse population) on women's knowledge about breast cancer and their breast self-examination behaviors.

## Methods

As part of a population-based randomized trial on different invitation methods for attending breast care classes in Tehran, a study was conducted to investigate Iranian women's views about breast cancer and their self-reported practice of BSE. The ethics committee of the Iranian Centre for Breast Cancer approved the study.

The study carried out in one of the central districts of Tehran that has five divisions with a total population of 310184 and was chosen because the district has a mixed population. The study population consisted of female population of aged 20 to 80 years (n = 117679). A cluster sampling was applied to include an enough sample size from each district's division in the study. Assuming that at best 40% of the women would perform BSE, it was calculated that a sample of 280 women aged 20 to 80 years from each division (a total of 1400 female) would allow detecting a 20% difference between performer and non-performer at 5% significance level. A study of this size has a power of 90%.

Data were collected via a structured questionnaire derived from the literature and our own previous studies [27-30]. A trained female nurse interviewed each respondent. An informed consent was obtained before conducting the interviews. The questionnaire consisted of 15 items on demographic characteristics, history of personal breast problems, family history of breast cancer, knowledge about breast cancer covering its symptoms, the screening methods and practice of BSE [Additional file 1]. Analysis included descriptive statistics and the Chi-squared test where necessary.

## Results

In all 1402 women were studied. The mean age of the respondents was 43.4 (SD = 14.4) years and most were married (85%). Ninety-four percent of the respondents indicated that "they did not have any personal breast disease" while the remaining 6% reported that "they had benign (5%) and malignant breast disease (1%)". The positive family history of breast cancer reported by 10% of women (n = 138). Of those with a family member with breast cancer (n = 138), 36% (n = 50) reported that their first-degree relatives (mother, sister and daughter) had breast cancer and the remaining 64% (n = 88) reported of the existence of breast cancer in their other family members. The demographic characteristics of the respondents are shown in Table 2.

**Table 2:** Demographic characteristics of the study sample (1402)

	**Number**	**%**
**Age groups (years)**		
20–29	258	18
30–39	354	25
40–49	334	24
50–59	220	16
60>	236	17
Mean (SD)	43.4 (14.4)	
Range	20–80	

**Educational status**		
Primary education	673	48
Secondary education	497	35
Higher education	232	17

**Marital status**		
Single	117	8
Married	1183	85
Widowed/divorced	102	7

**Personal history of breast problems**		
Yes	84	6
No	1318	94

**Family history of breast cancer**		
Yes	138	10
No	1264	90

When the respondents were asked about breast cancer in Iran, 64% (n = 894) said that "they have heard about the disease" and 61% (n = 851) believed that "breast cancer is relatively a common disease among women in Iran". The respondents' knowledge of breast cancer symptoms was also studied. Most women (44%) perceived a painless mass as a breast cancer symptom.

Overall the study findings indicated that 61% of the respondents knew about breast cancer screening methods: 31% knew about breast self-examination, 21% about clinical examination and 9% about mammography. The remaining 39% claimed that they know nothing about breast cancer screening methods. Most respondents (48%) said that electronic media (television 34% and radio 14%) were their source of information.

When the respondents were asked about breast self-examination, 37% (n = 520) reported that 'they practice breast self-examination'. Only 17% of women said that 'they do regular breast self-examination'. When it was investigated to find out women's reasons for not doing breast self-examination, 64% claimed that 'they do not know how to do it'. Table 3 shows the respondents' knowledge of breast cancer and self-reported practice of breast self-examination.

**Table 3:** Respondents' knowledge of breast cancer and self-reported practice of breast self-examination (n = 1402)

	**Number**	**%**
**Have you heard about breast cancer in Iran?**		
Yes	894	64
No	508	36

**What do you think about breast cancer in Iran**		
It is a rare disease among women	200	14
It is relatively a common disease among women	851	61
I don't know	351	25

**Breast cancer symptoms**		
Painless mass	618	44
Painful mass	183	13
Nipple retraction	72	5
Breast pain	223	16
Breast discharge	81	6
Bloody discharge	118	8
Breast asymmetry	20	2
Don't know	87	6

Knowledge about screening programs*		
Heard of BSE	505	31
Heard of clinical examination	337	21
Heard of mammography	144	9
Knew nothing	626	39

Source of information*		
Radio	199	14
Television	487	34
Printed materials	191	13
Friends	284	20
Others (physicians, family, etc.)	271	19

**Frequency of self-reported BSE**		
Regularly (monthly)	238	17
Occasionally	282	20
Never	882	63

* The respondent could choose more than one response category. The percentages are based on total responses and not total number of the respondents.

Further analysis of the data carried out to investigate about the relationship between the performance of breast self-examination and demographic and other studied variables. The findings indicated that performing breast self-examination is significantly related to: age (χ^2 ^= 28.9, P = 0.00006), marital status (χ^2 ^= 10.3, P = 0.03), education (χ^2 ^= 73.1, P < 0.00001), knowledge about breast cancer (χ^2 ^= 153.4, P < 0.00001), and knowledge about breast cancer screening programs (χ^2 ^= 254.9, P < 0.00001), but not to personal (χ^2 ^= 3.4, P = 0.2) and family (χ^2 ^= 0.2, P = 0.7) history of breast problems. In fact the results suggest that women aged 40 to 59, married, with higher education and more informed about breast cancer were more likely to perform BSE.

## Discussion

This population-based study carried out to investigate about Iranian's female knowledge of breast cancer and their BSE practicing behavior. The study sample was relatively a representative sample of the study population except in marital status (with a larger proportion of married women) and in education (with a smaller proportion of educated people). This was due to the fact that basically Iran has a young population (about 50%) and that the younger age females (mostly single and educated) were not included in the study.

In this study 64% of respondents said that they knew about breast cancer and 17% of whole sample indicated that they were performing regular monthly BSE. Lower rates of BSE performance have reported from developing countries. A study from Saudi Arabia found that only 30.3% of the women had heard about breast self-examination and 18.7% reported they practiced BSE within the previous year [31]. Another study from Nigeria demonstrated that women lacked enough knowledge about breast cancer and only 34.9% claimed to ever-practiced BSE [32]. While a study of BSE behavior among Chinese immigrant women living in San Francisco indicated that 80.9% reported having heard of BSE but only 53.9% of the women had performed BSE during the past year [33]. Comparing the figures with that of developed countries clearly suggests that there are obvious differences. It has been shown that 75% of the women conduct BSE in the United States and its adequate quality was rated in 27%. Also higher duration, frequency and quality of BSE were predictors of further diagnostic investigations [34]. Similarly an Austrian study reported that about 31% of women examined their breasts thoroughly [35].

Although 'painless mass' was the most reported symptom of breast cancer by women, the study results indicated that women had inadequate knowledge about this breast cancer symptom. Only 44% of women said that painless lump is a common symptom of breast cancer. The remaining 46% indicated that they 'don't know'. The figures even were lower for other symptoms. For instance, only a few proportions of women knew that nipple retraction (%5) and bloody discharge (6%) are warning signs of breast cancer. About screening programmes the awareness knowledge was also very inadequate: only 21% and 9% have respectively heard about breast clinical examination and mammography. This is consistent with other studies from developing countries and women from minority ethnic groups [32,36], whereas a study from U.K indicated that 70% of women were well aware of 'painless lump' and able to identify these symptoms in their breast self-examination [37]. A recent publication from U.K reported a significant lack of the prerequisite knowledge and confidence to detect a breast change among older women aged 63 to 73 years [38].

However although cultural differences might contribute to such variations, the role of some other underlying factors on breast health awareness in women should not be neglected. Studies have shown that formal training programs have profound effects on regular BSE performance with a correct technique [39,40]. A study from Turkey showed that theoretical educations on breast cancer awareness and BSE training were effective even in illiterate and low-educated women [41].

The main sources of information about breast cancer were 'mass media' (48%) and followed by friends (20%). In most developing countries mass media are governed by the 'states' and using them would help to increase awareness about breast cancer. Usually due to some religious and cultural reasons such programs receive less attention in public media. Furthermore a very low proportion of women indicated that they have received any information from their doctors. Indeed many women (64% of non performers) stated that they 'do not know how to do BSE'. Primary health care professionals would play an important role in conveying correct information regarding breast cancer [42]. It is argued that ignorance regarding incidence, outcome and risk of breast cancer makes it unlikely that at risk females could currently make informed decisions on a range of breast issues [43].

Statistical analysis indicated that performing BSE was significantly related to age, marital status, education, knowledge of breast cancer and its screening programs. It has been shown that women with less formal education more likely had inadequate knowledge about breast cancer that inversely influenced their breast cancer screening behaviors [44]. Another study based on health belief model showed intentions to do BSE among Turkish women is associated with "informed about breast cancer and having health insurance" [45].

This study did not show a significant association between personal and family history of breast cancer and performing BSE as compared to women without personal and family history of breast problems. In contrast, studies have shown that women with a family history of breast cancer may perform excessive BSE. Designing appropriate interventions would enhance the women's confidence in their ability to do BSE effectively and reduce worries regarding their breast cancer risk [46]. However, since women having any breast problems are expected to be different in their perceptions, knowledge and attitude from those having benign or malignant breast diseases, the future studies should include only women without any breast problems.

## Conclusion

Although this descriptive study has its own limitations, for the first time it provides useful information that could be utilized by both researchers and those involved in public health programmes. The findings indicated that the women awareness of breast cancer warning signs (painless lump, retraction of nipple, and bloody discharge) and effective screening methods i.e. clinical examination, and mammography were very inadequate. Thus, health education programmes to rectify the lack of women awareness is urgently needed. Indeed the focus of primary health care providers should be to raise awareness about breast care among women and to encourage them to report any unusual changes in their breasts to their family or care physicians.

## Abbreviations

BSE: Breast Self Examination

## Competing interests

The authors declare that they have no competing interests.

## Authors' contributions

AM designed the study, analyzed the data and wrote the paper. MV contributed to the data entry, literature review and writing-up process. IH and AMH contributed to the study design and analysis. AS contributed to the data collection and the data entry. FK, ME, SH and SJ contributed to the study design and helped in writing the manuscript. All authors read and approved the final manuscript.

## Supplementary Material

Additional File 1 Breast cancer: knowledge, screening methods and breast self-examination survey. This is a short questionnaire assessing women awareness on breast cancer and its screening methods. It also asks whether women perform breast self-examination or not. Click here for file
