# Supplementary material for: Breast cancer in Iran: need for greater women awareness of warning signs and effective screening methods
Source: Asia Pac Fam Med. 2008 Dec 20;7(1):6. doi: 10.1186/1447-056X-7-6 (PMC2628874; doi:10.1186/1447-056X-7-6)
Supplement: Additional File 1 — Breast cancer: knowledge, screening methods and breast self-examination survey. This is a short questionnaire assessing women awareness on breast cancer and its screening methods. It also asks whether women perform breast self-examination or not. [file 1447-056X-7-6-S1.doc]

Breast cancer: knowledge, screening methods and breast self-examination survey

# Iranian Centre for Breast Cancer (ICBC)

No.: ……………

Date: …...………

# DEMOGRAPHIC DATA AND HISTORY

| **1. Date of birth…………………………** |  |  |
| --- | --- | --- |
|  |  |  |
| **2. Level of education** |  |  |
|  | □ | Illiterate |
|  | □ | Primary |
|  | □ | Secondary |
|  | □ | Higher education |
|  |  |  |
| **3. Marital status** |  |  |
|  | □ | Single |
|  | □ | Married |
|  | □ | Divorced/widowed |
| **4. Employment status** |  |  |
|  | □ | Housewife |
|  | □ | Employed |
|  | □ | Student |
|  | □ | Retired |
|  |  |  |
|  |  |  |
| **5. History of breast problem** |  |  |
|  | □ | Yes |
|  | □ | No |
| **6. Type of breast problem** |  |  |
|  | □ | Breast cancer |
|  | □ | Breast pain |
|  | □ | Benign disease |
|  | □ | Others |
|  |  |  |
| **7. Family history of breast cancer** |  |  |
|  | □ | Yes |
|  | □ | No |
| **8. If yes, please indicate:** |  |  |
|  |  | □ mother □ sister □ daughter □ grandmother □ aunt □ others |

KNOWLEDGE OF BREAST CANCER AND SCREENING METHODS

| **9. Have you ever heard about breast cancer in Iran?** |  |  | | | |
| --- | --- | --- | --- | --- | --- |
|  | □ | Yes | | | |
|  | □ | No | | | |
|  |  |  | | | |
| **10. What do you think about breast cancer in Iran?** |  |  | | | |
|  | □ | It is a rare disease among women | | | |
|  | □ | It is relatively a common disease among women | | | |
|  | □ | I don’t know | | | |
|  |  |  | | | |
| **11. Which of the followings is a common symptom of breast cancer?** |  | **True False I don’t know** | | | |
|  | - | Painless mass | □ | □ | □ |
|  | - | Multiple masses | □ | □ | □ |
|  | - | Nipple retraction | □ | □ | □ |
|  | - | Breast pain | □ | □ | □ |
|  | - | Breast discharge | □ | □ | □ |
|  | - | Breast asymmetry | □ | □ | □ |
|  | - | Bloody discharge | □ | □ | □ |
|  |  |  |  |  |  |

| **12. What should women do for early detection of breast cancer?** |  |  |  |  |  |
| --- | --- | --- | --- | --- | --- |
|  | □ | Monthly breast self examination | |  |  |
|  | □ | Annual breast examination by a doctor | | |  |
|  | □ | Both |  |  |  |
|  | □ | None |  |  |  |
|  | □ | I don’t Know |  |  |  |
| **13. Would you please indicate your source of information about breast cancer?** |  |  |  |  |  |
|  | □ | Radio | | | |
|  | □ | Television | | | |
|  | □ | Printed materials | | | |
|  | □ | Friends | | |  |
|  | □ | Family | | |  |
|  | □ | Physicians | | |  |
|  | □ | Others | | |  |
|  |  |  | | |  |

**PRACTICE OF BREAST SELF-EXAMINATION**

| **14. Do you examine your breasts?** | □ | No |  |
| --- | --- | --- | --- |
|  | □ | Yes, occasionally |  |
|  | □ | Yes, monthly |  |
| **15. Reasons for not doing BSE** |  |  |  |
|  | □ | I do not know how to do it |  |
|  | □ | I forget |  |
|  | □ | Fear of finding a breast mass |  |
|  | □ | It takes time |  |
|  | □ | Others |  |

# Thank you for your cooperation.
